# An auto-adaptive optimization approach for targeting nonpoint source pollution control practices

**DOI:** 10.1038/srep15393

**Published:** 2015-10-21

**Authors:** Lei Chen, Guoyuan Wei, Zhenyao Shen

**Affiliations:** 1 State Key Laboratory of Water Environment, School of Environment, Beijing Normal University, Beijing 100875, P.R. China

## Abstract

To solve computationally intensive and technically complex control of nonpoint source pollution, the traditional genetic algorithm was modified into an auto-adaptive pattern, and a new framework was proposed by integrating this new algorithm with a watershed model and an economic module. Although conceptually simple and comprehensive, the proposed algorithm would search automatically for those Pareto-optimality solutions without a complex calibration of optimization parameters. The model was applied in a case study in a typical watershed of the Three Gorges Reservoir area, China. The results indicated that the evolutionary process of optimization was improved due to the incorporation of auto-adaptive parameters. In addition, the proposed algorithm outperformed the state-of-the-art existing algorithms in terms of convergence ability and computational efficiency. At the same cost level, solutions with greater pollutant reductions could be identified. From a scientific viewpoint, the proposed algorithm could be extended to other watersheds to provide cost-effective configurations of BMPs.

Nonpoint source (NPS) pollution has recently been regarded as the major contributor to worldwide water quality deterioration, and best management practices (BMPs) are implemented to reduce the release of NPS pollutants^1^,^2^. Due to the complexity of watershed processes, the design of BMPs at the watershed scale is inherently a multi-objective optimization problem^3^. Generally, BMPs are divided into structural practices, in terms of filter strips, parallel terraces and grassed waterways, and non-structural practices, such as tillage operation and nutrient management. Determining the types, number, locations and configurations of BMPs are therefore some of the most serious challenges facing both watershed managers and the public.

Optimization algorithms have been widely used in the optimal design of BMPs^2^,^4^,^5^,^6^. Many algorithms, such as simulated annealing, ant colony optimization, and differential evolution, have been proposed, and theoretical information regarding these approaches has been summarized by Zecchin^7^. The appeal of these algorithms for NPS pollution problems is multifaceted. First, these algorithms are global optimizers and able to deal with multi-objective, high dimensional, nonconvex and constrained problems. Second, gradient or Hessian information about the objective are not required so these algorithms can solve discrete, continuous or stochastic problems^8^,^9^. Among these algorithms, genetic algorithms (GAs), originally proposed by Holland^10^, and their extensions, such as non-dominated sorting genetic algorithms (NSGAs)^11^, have been commonly used due to their iterative and parallel subpopulation features. However, optimal designs of BMPs in real-world situations can be extremely difficult to solve^12^. The technical complexity and computational burden involved are the two greatest barriers to the adoption of these previous approaches^13^.

Firstly, optimization algorithm itself may be a barrier. Generally, the performance of heuristic algorithms, in terms of the process of natural evolution, their computational ability, and their efficiency in locating Pareto-optimality solutions, are determined by the parameters settings (e.g., population size, number of generations, crossover probability, and mutation probability in the case of GAs)^14^. Despite their critical importance, defining these parameters is often difficult and subjective, typically involving trial-and-error sensitivity analysis^2^,^7^,^14^. However, such models are extremely computationally difficult to solve, especially in a series of watershed simulations. In addition, because many optimization parameters are inter-dependent^15^, there is no well-accepted theory or methodology for the calibration of those parameters; furthermore, watershed processes are highly complex and comprise a network with nonlinear dynamics^16^. Therefore, a specific set of parameters can only be selected for a particular watershed and cannot be extended to other watersheds due to the varying characteristics of environmental problems^15^.

In addition, the fixed values of parameters may be another potential shortcoming of the evolutionary process. Previously, fixed-valued parameters have been widely used to capture the characteristics of a watershed optimization problem. For example, the use of greater crossover and mutation probabilities allows GA to search large solution spaces, in terms of global convergence and the diversity of the optimal solutions, whereas smaller parameters would benefit localized searches^2^,^17^. In addition, larger populations or more generations would yield better Pareto-optimality solutions by increasing the parallel subpopulations but would also delay convergence or increases the computational time^14^. In this sense, the self-adaptation of these parameters as inherent to the optimization variables or hybridization processes might be an effective way to enhance the overall performance of the optimization algorithm^13^,^15^. Despite these efforts, there is still no systemic study providing insights into how to navigate the values of these parameters to find the final Pareto- optimization solutions.

Therefore, the primary goals of this study are 1) to modify the optimization parameters into an auto-adaptive pattern; and 2) to establish a new framework for the optimal design of BMPs at the watershed scale. Section of methodology describes the proposed framework in detail, and Section of results demonstrates an applied case study in the Daning River watershed (108°44′–110°11′E, 31°04′–31°44′N), in the central part of the Three Gorges Reservoir Area (TGRA), China.

## Results

### Evolutionary results of the GA parameters

**Figure 1:**
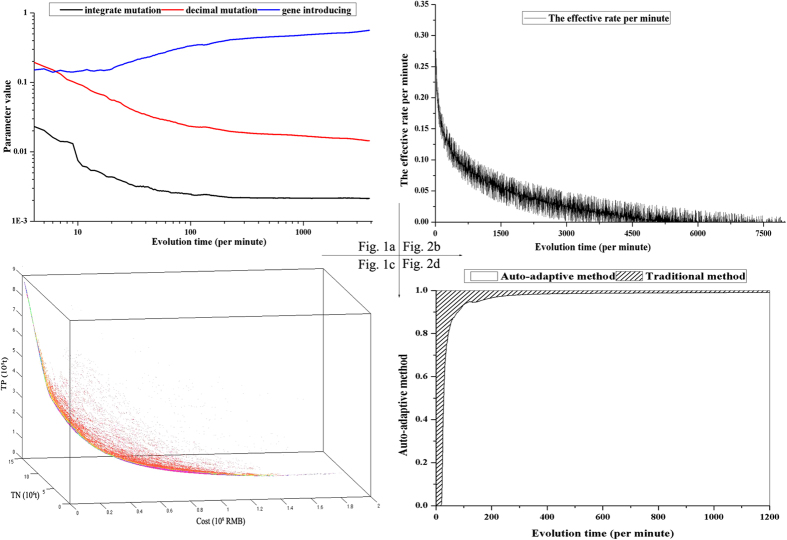
The evolutionary process of the auto-adaptive optimization parameters. Note: (**a**) The evolution process of integrate mutation, decimal mutation and gene introducing parameter; (**b**) The evolution process of the effective rate per minute; (**c**) The evolution process of auto-adaptive pollution size; (**d**) The comparison between auto-adaptive and traditional optimization algorithms. These figures were drawn by the Matlab 2012a and Originlab softwares.

The evolutionary processes of the genetic parameters are shown in Fig. 1a,b. As illustrated in Fig. 1a, the values of these parameters changed significantly in the first 10 minutes. Specifically, the integer and decimal mutation probabilities decreased from their default maximum values (set at 2) to 0.01 and 0.10, respectively. As the algorithm processed, the integer mutation probability changed gradually, but the decimal mutation probability changed more substantially. During the following evolutionary processes, these parameters reached relatively stable states in 87 minutes and 212 minutes, respectively. Conversely, the introduction probability increased noticeably from 0.14 to 0.45 during the first 300 minutes, but its rate of increase leveled off gradually in the following 3000 minutes. Unlike the mutation operation, an increase in the introduction probability always results in better reproduction from the mating pool, and thus, this variation would lead to good convergence. Finally, the optimal front was found when the integer mutation, decimal mutation and introduction probabilities were 0.002, 0.014 and 0.56, respectively.

Instead of using the traditional concept of generation, the effective rate, which was quantified as the generation rate of the Pareto-optimality chromosomes and total chromosomes per minute, was tested as the genetic termination condition. As illustrated in Fig. 1b, the effective rate fluctuated during a few periods due to the stochastic nature of heuristic algorithms but showed an obvious decreasing trend. As the algorithms progressed, the effective rate decreased significantly from 0.37 to 0.20 in 20 minutes, and changed gradually from 0.2 0 to 0.10 in the following 300 minutes. Then, the effective rate changed very slowly and reached the final value (0) in 4000 minutes, indicating that few Pareto-optimality chromosomes would be generated in the following few successive periods. This result confirmed that the effective rate could be used as the genetic termination condition of the optimization algorithms. In this sense, watershed managers would not need any knowledge of the optimization problem for automatic termination^18^.

As shown in Fig. 1c, the increasing population size, in terms of the number of points (chromosomes), approaches to the origin. During the evolutionary process, the population size was further increased from the default 1-chromosome to the final 26,820-chromosomes. The entire evolutionary process required approximately 300 minutes of computational time on a desktop personal computer (Centrino Duo processor running at 2.8 GHz). However, if the algorithms processed further, the population size would increase gradually to 56,462 in the following 1200 minutes and this ever-growing population would increase the computational burden.

### Evolutionary results of the Pareto-optimization front

**Figure 2:**
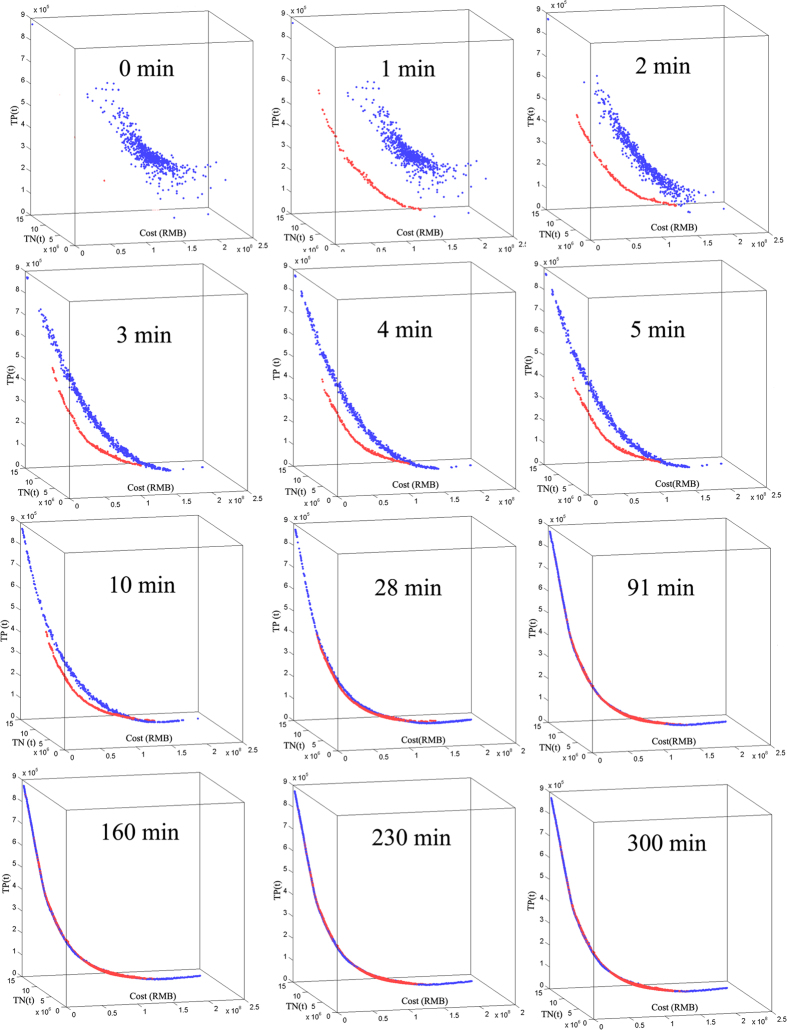
The evolutionary process of the Pareto-optimality front. Note: Red point and blue point represents the proposed auto-adaptive optimization algorithm and traditional NSGA-II, in which the points in the Pareto front represent the number of optimal solutions, and axis distance between points represents the magnitude of objective changes. These figures were drawn by the Matlab 2012a software.

As shown in Fig. 2, the shape of the Pareto trade-off front obtained by the proposed method (red point) shifted from relatively scattered points to a smooth curve due to the increasing population size. The final Pareto-optimality front indicated average reductions of N and P loads from 43.74%–93.38% and 49.74%–88.83%, respectively. In our previous studies^19^,^20^, the average concentrations of TN and TP at the Wuxi station were quantified as 0.82 and 0.13 mg/l, respectively, indicating a further requirement of 39.02% and 23.07% load reductions from the baseline. Figure 2 indicates that the optimal watershed-scale BMP configurations would be disproportionate to the intended objective of maintaining the water quality provisioning services in the TGRA. Furthermore, fertilizer management and conservational tillage are continuously included in each optimal solution on the lower edge of the front curve, even from an early evolutionary point. However, structural BMPs are observed on the higher edge of the front curve, where those expensive solutions are located. These results indicated that effective watershed management could first can be achieved through a combination of non-structural BMPs. To reach further reductions of NPS-N and -P, a combination of structural BMPs is suggested for the entire catchment.

## Discussion

Generally, fixed-value parameters are used for the traditional NSGA-II method^2^,^7^,^12^. Therefore, a trial-and-error sensitivity analysis was conducted in this study to compare the proposed auto-adaptive and fixed-value parameters. Based on the results, as the mutation probability decreased from 1 to 0.01, the Pareto-optimality front approached the origin, however, when this parameter was further decreased from 0.01 to 0, the Pareto-optimality front moved away from the origin. Similar shifts have also been found in several previous studies^2^,^7^,^14^. Finally, the best solutions of NSGA-II were found when the mutation probability was set to 0.01. However, Fig. 1a indicates that the auto-adaptive mutation probability would evolve automatically from large values to small values during the evolutionary process. These variations would allow the optimization algorithm to search a large solution space (global search) at first, and then focus on smaller variable spaces (local search) afterwards^21^. The change from a global to local search could be due to the dynamic variations of these genetic parameters, as well as the decreasing benefits of variable spaces. Therefore, if the mutation probability were set to a fixed value of 0.01, the Pareto-optimality front would eventually move away from the origin. A consistent pattern in the shift of the Pareto-optimality front was also found for the crossover probability (introduction probability). In this sense, the use of the genetic parameters as an inherent portion of the decision variable provides an effective method to enhance the periodic evolutionary process of the optimization algorithms^13^.

In addition, more optimal solutions were found when the generation and population size of NSGA-II were fixed at 1,500 and 700, respectively. Conversely, as shown in Fig. 1b,c, the values of the auto-adaptive population and effective rate would be moderated in an attempt to obtain better Pareto-optimality solutions. Generally, any increase in these parameters would provide more individuals, and a longer evolutionary time, which would result in a higher probability of obtaining better offspring. As shown in Fig. 1b, the effective rate of auto-adaptive evolution decreased from 0.37 to 0.10 in 300 minutes. Those 300 minutes also represent the computational time required to calculate 1,500 generations using the traditional NSGA-II method. As the effective rate further decreased to 0, the Pareto-optimality front approached the origin and more effective individuals were generated (Fig. 1c). However, only one parameter was changed in this study, and the other three parameters were fixed at their default values during the sensitivity analysis. Therefore, if the population size were fixed at a larger value, more generations would be required for the NSGA-II as further computational time would be needed to show considerable changes in those objective functions^13^. In this sense, the termination criteria of Pareto-optimality were tightened based on the principles of the naturally-random evolution of these GA parameters.

Furthermore, the optimal configuration of BMPs is almost certainly case-specific when using traditional NSGA-II^15^, i.e., dependent upon the type of BMPs and the targeted pollutant. Instead, the proposed auto-adaptive method provided a very robust and parsimonious method within a specific computational budget. In practice, if the user is dissatisfied with the current solution, they could vary the effective rate (Fig. 1b) and give this auto-adaptive algorithm more computational time.

Generally, two points should be considered in the choice of an optimization algorithm: convergence ability and computational efficiency^17^,^22^,^23^. In this study, the evolutionary progress of the Pareto-optimal front is plotted in Fig. 2, in which the proposed method and the traditional NSGA-II method are represented by red and blue points, respectively. As illustrated in Fig. 2, in the first 30 minutes, the Pareto trade-off front of the proposed method was closer to the origin point than that of the traditional NSGA-II method, which could be due to the more effective reach of the proposed method by eliminating portions of the ineffective variable space from even the initial successive search. As shown in Fig. 1b, the greatest effective rate could be observed at approximately 1 minute, indicating that an effective search had been conducted even when the auto-adaptive algorithm began. Thereafter, the proposed Pareto-optimality front approached the origin more slowly, which prevented convergence to previous local optima. Finally, a smoother curve was generated by the proposed auto-adaptive method due to its larger population size, in terms of more solutions on the Pareto-optimality front.

As illustrated in Fig. 2, the final computational time was 340 and 300 minutes for the proposed method and the traditional NSGA-II method, respectively. Thereafter, all Pareto-optimality solutions per minute were mixed and compared in the mating pool. The Pareto concept offered the necessary logics for the prioritization of these two methods in the evolutionary process^2^,^7^,^14^. As shown in Fig. 1d, the traditional NSGA-II method initially generated more Pareto-optimality solutions due to its greater initial population size. As the algorithm progressed, equal percentile (50%) could be observed at 26 minutes for the two methods, which indicated that the proposed method generated as many Pareto-optimality solutions as the NSGA-II method in a very short time (less than 10% of the computational time). If the algorithm continues for a longer time, more effective solutions, with better convergence would be obtained by the proposed method. In this minimization problem, a Pareto-optimality front with greater fitness function values also indicates better convergence ability^2^,^5^,^24^,^25^. As shown in Fig. 2, on average, 8% and 6% greater reductions in N and P can be obtained at the same cost by using the proposed method rather than the traditional NSGA-II. This confirmed that auto-adaptive optimization has a better search capability (the ability to find Pareto-optimal solutions), and is more computationally efficient than the traditional NSGA-II method.

**Figure 3:**
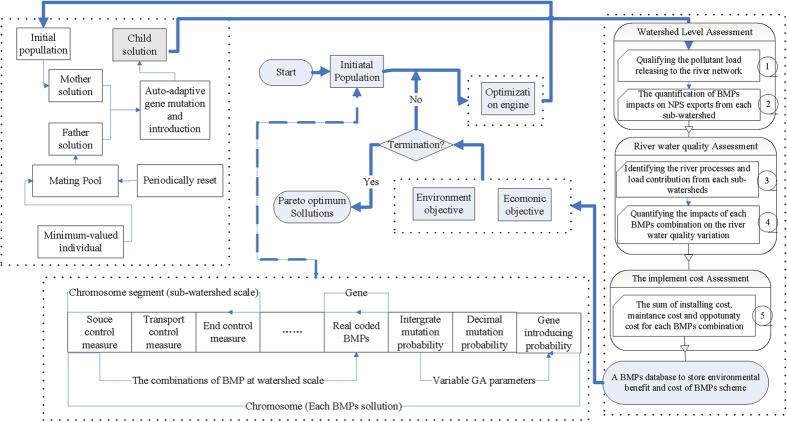
The framework of the proposed auto-adaptive optimization algorithm.

In addition to convergence efficiency, the diversity of the Pareto-optimality solutions is another important factor in characterizing the optimization algorithms^2^,^7^,^14^. As illustrated in Fig. 2, more effective solutions were generated by the proposed method due to its larger population size, which can store those increasingly effective solutions. Interestingly, the Pareto-optimality fronts of these two methods were similar at lower costs; this could be because the proposed method, like the NSGA-II, is characterized by the effective retention of the less crowded mother solutions as the main bodies of child chromosomes during the evolutionary process^2^,^21^. However, the proposed method outperformed NSGA-II at higher costs in exploring a wider variable space and locating more promising regions. This indicated that the auto-adaptive algorithm increased the diversity of the individuals on the higher edge of the Pareto-optimality front (Fig. 3). The auto-adaptive algorithm is designed to overcome the inherent shortcomings of traditional NSGA-II. To our knowledge, this study is the first use of an auto-adaptive strategy in an actual application of optimal BMPs configurations. Overall, the results suggested that this strategy works well, even for structural BMPs, for which many researchers have reported difficulty in finding feasible solutions due to their smaller variable space^26^,^27^,^28^. Finally, although the auto-adaptive optimization was developed for direct application to NSGA-II, it is also appropriate for other similar optimization methods.

## Methodology

### Study watershed

In this study, the Daning river watershed (108°44′–110°11′E, 31°04′–31°44′N) was selected as the study area. The Daning river is located in the Wuxi country of the district of Chongqing municipality, in the central part of the Three Gorges Reservoir Area (TGRA), China. It originates from northern mountains and travels a basin that includes forests and agricultural plains, and eventually influxes to the Yangtze River. The study watershed, with a drainage area of 4,426 km^2^, is a mixed land use area, 22.2% of which is covered by agricultural land (paddy land and dry land), 65.8% by forest and 11.4% by grassland. The main soils are 14.65% by purple soil, 11.0% by yellow soil, 26.5% by yellow-brown soil and 16.9% yellow cinnamon soil. The humid subtropical climate features this watershed, with the annual average temperature of 16.6 °C and precipitation being around 1124 mm. Due to an increase in fertilizer inputs, the P and N concentrations have increased greatly over the last 10 years, which has already resulted in not only significant on-site phytoplankton growth in the region, but also causes off-site problems related to downstream eutrophication to TGRA. Therefore, we focus on minimizing P and N loads.

### Description of the framework

Figure 3 illustrates the integrated framework for the optimal designs of watershed BMPs. An auto-adaptive algorithm was initiated using the NSGA-II to enhance the computational efficiency of the entire optimization procedure. Each objective was then evaluated using the appropriate tools: 1) a watershed model, for quantifying the pollutant loads under scenarios with and without BMPs; and 2) an economic module, for calculating the implementation cost of each scenario.

### Initial population including the chromosome for BMPs combinations at the watershed scale

As a geographically connected unit, a watershed consists of a stream network and its corresponding sub-watersheds^16^,^19^. The river network can be extracted from a digital elevation map (DEM) and connected to each sub-watershed using the hydrology module in ArcGIS^29^. The sub-watershed is then broken into several smaller cells or hydrologic response units (HRUs) that consist of a homogeneous slope, land use, and soil type. Typically, non-structural BMPs are targeted to a certain land use, whereas structural BMPs are executed within smaller spatial units. Rather than using several dozen cells, the entire Daning watershed was subdivided into almost 80 sub- watersheds, and each sub-watershed was further divided into smaller HRUs approximately the same spatial dimensions as the relevant structural BMPs. Such a partitioning is essential to achieve the desired spatially explicit resolution within a fairly simple formulation.

In addition, non-structural BMPs are often implemented as source control measures, and structural BMPs are installed in certain stream segments as transport control measures^12^. Specifically, detention ponds and wetlands are designed at the drainage outlet as end control measures^24^. In this sense, different BMPs were grouped into three categories at the watershed scale (Table 1). At the sub-watershed scale, the aggregated configuration of the BMPs was represented by randomly sampling from these categories and real-coding the selection as a chromosome segment. Specifically, the integer part and decimal part of gene were used to represent the type and corresponding parameters of each categorized BMP. As illustrated in Fig. 3, the configuration of BMPs at the watershed scale was then real-coded as a population of chromosomes. In this sense, three genetic parameters, including integer mutation, decimal mutation, and probability of introducing, were added as genes at the end of each chromosome as inherent part of the optimization variables.

### Watershed, river water quality, and cost level assessments

#### Preparation of the watershed model

In this study, the watershed processes and the pollutant loads released from each spatial unit were obtained from our previously calibrated Soil and Water Assessment Tool (SWAT)^30^. First, the SWAT model was calibrated and validated for the simulated flow, sediment, nitrogen (N) and phosphorus (P). More details regarding the calibration process can be found in previous studies^31^,^32^,^33^. Second, the calibrated SWAT was used to simulate the watershed’s hydrological and water quality responses during the baseline scenario (without BMP implementation). A 10-year period (2000–2009) was modeled to isolate the variability of climate, land use, crop rotations and runoff regime which might mask the effects of BMPs. Third, the N and P load reductions during the BMP scenario were quantified at the Wuxi monitoring station using straightforward changes in the model parameter and updates to the management practices or land use^24^,^34^. In consideration of the temporal scale and data availability, the environmental benefits of each BMP configuration were quantified as the average load reductions of N and P at the Wuxi station during the simulation period of 2000–2009.

#### Quantification of the implementation cost

The Farm-level Economic model (FEM) was used as economic estimators to quantify the implement cost of individual BMPs, in which the total implementation cost consists of the installation cost and the maintenance cost.

(i) Installation cost:
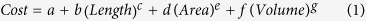
where *Length*, *Area* and *Volume* represents the corresponding physical parameters of the BMPs; and *a, b, c, d, e, f,* and *g* denotes the empirical coefficients in Equation 2 (the default values are shown in Table 2).

(ii) Maintenance cost:

The maintenance expenditure was evaluated as a percentage of the installation cost (Table 2). This annual expenditure was then projected as a present net value:
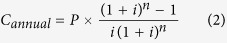
where *C*_*annual*_ and *P* represents the present net value and the annual maintenance cost, respectively; and *i* and *n* denotes the interest rate (5% in this study) and designed lifetime (Table 2), respectively.

Finally, the total cost 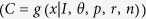 was quantified by summing the implementation cost of each individual BMP throughout the watershed.

#### Environmental/economic objectives

In this study, environmental and economic considerations were incorporated as two optimization objectives. The proposed fitness functions were then expressed as follows:
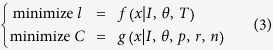
where *l* and *C* represents the load reduction of a certain targeted pollutant and the implementation cost over the period *T*, respectively; *f* and *g* represents the watershed model and economic module, respectively; *x* denotes the combinations of BMPs at the watershed scale; and *I*, *θ*, *p*, *r* and *n* represents the type, parameter, cost, benefit and lifetime of the BMPs, respectively.

A BMPs database, which stores the pollutant load reductions and implementation costs of each BMPs scheme, was developed from a number of consecutive SWAT and economic model runs^32^,^33^. Using the process described below, this database provides necessary inputs into the optimization engine to decrease the computational time.

### Development of optimization engine

#### The description of initial NSGA-II

In this study, the NSGA-II method was chosen because it has gained popularity in BMPs design and can overcome issues of high computational complexity^35^. The major difference between the NSGA-II and other GAs are as follows: 1) the NSGA-II adopts a crowding distance to measure the density of individuals; 2) the fast non-dominated sorting approach is applied as a better elitist-keeping strategy; 3) the elitist crowded comparison guides the selection process at various stages towards a uniformly spread-out Pareto-optimality front^36^. Each BMPs scheme underwent genetic operations, and the algorithm terminated if a specified range of Pareto-optimality solutions had been generated.

#### The incorporation of the auto-adaptive pattern

A variant of the popular NSGA-II method, which is coded in the Matrix Laboratory R2012b GA toolbox, was used to navigate the evolutionary process of each auto-adaptive parameter. As illustrated in Fig. 3, these generic parameters were added as genes at the end of each chromosome as the inherent optimization variable. The definitions of these parameters were based on the following claims:

**Claim 1**: The introduction probability and mutation probability are defined as the extensions of traditional concepts. The introduction probability, which is also called recombination, defines the number of genes in the mother solution that are combined with a corresponding fraction of genes in the paired father chromosome. Mutation probability indicates the number of genes that would be randomly changed from their current state in producing the child chromosome.

Initially, these genes were given large values to allow the random search to occur within a larger region of variable spaces^2^,^21^. As shown in Fig. 3, pair of chromosomes (parents) was selected for mating during the reproduction process. The mother chromosome was drawn from the existing chromosomes using the tournament algorithm^36^. To increase the diversity of Pareto-optimality solutions, the mother chromosome with the larger crowding distance was used as the main body of the child chromosome. The father chromosome was taken from the mating pool and used as the source of gene introduction. In the following hybridization process, the mother chromosome was altered from its normal distribution magnitude to generate the child chromosome. In addition, appropriate parameters for the candidate mother chromosome were selected and changed slightly to generate the parental similarity parameters. In this sense, the generic genes of the parent chromosomes were retained and used to produce the offspring parameters through biological evolutionary processes.

**Claim 2**: The auto-adaptive heuristic search is not a fixed-population-based algorithm. Initially, only one chromosome was randomly generated, and the minimum-valued chromosomes (at least one objective) were added to the mating pool as father (seed) chromosomes. In successive iterations, every child chromosome was considered as a potential seed and compared with the existing chromosomes in the current population *Xs*. If no other dominant chromosomes existed, the current child chromosome would itself become a species seed in the population *Xs*. This procedure was repeated for all child chromosomes and those dominant chromosomes were eliminated from *Xs*. Theoretically, any update in the Pareto-optimality candidate chromosomes yields a larger population size. The subsequent evolutionary process was then divided into more parallel sub-simulations that simultaneously evolve. This automatically increasing population size (*Xs*) can be used to save increasingly desirable chromosomes and therefore to locate more Pareto-optimality solutions. To reduce the computational time due to the increasing population size, the dichotomy approach was adopted to rank each child chromosome.

Correspondingly, a growing mating pool was used for the elite reservation strategy (Fig. 3). If an effective child chromosome was generated, the mother chromosome was selected as elite and put into the mating pool as a father chromosome. When a father chromosome was chosen from the mating pool, the default value of its reproduction ability was defined as 1. During the reproduction process, this value would be increased by 1 if one dominant child was generated, and decreased by 1 if no dominant child was generated. A new father chromosome would be chosen from the mating pool if the value of its predecessor had decreased to 0. As a greedy algorithm, the minimum-valued and best chromosomes, once identified, are never updated^15^. However, other chromosomes in the mating pool would be reset periodically to reduce the computational time.

## Final Pareto optimal solutions

The traditional generation concept was replaced by the periodical statistics of this auto-adaptive algorithm. Iterative statistics were performed, which repeatedly applied statistical analysis during each successive period (i.e., per minute). To prevent convergence to previous local optima, portions of the variable spaces were eliminated from the successive searches. In this sense, this algorithm searches globally at the beginning of the search, and transitions to local searches as the optimization progresses. The desirable convergence was confirmed, and the algorithm terminated when no more dominant chromosomes were generated during a few successive periods. The default termination condition would be reached if the number of Pareto-optimality chromosomes exceeded the product of the population size and objective numbers. Otherwise, a maximum effective rate, designed as an algorithm input rather than as a parameter, should be set according to the available (or desired) computational time^15^.

## Additional Information

**How to cite this article**: Chen, L. *et al*. An auto-adaptive optimization approach for targeting nonpoint source pollution control practices. *Sci. Rep.* **5**, 15393; doi: 10.1038/srep15393 (2015).

## Figures and Tables

**Table 1:** Categories of different BMPs at the sub-watershed scale.

**Type of BMP**	**Specific BMP examples**	**Category**
Non-structural BMPs	Conversion of farm land into forest/grass	Source control measure
	Fertilization reduction	Source control measure
	Conservation tillage	Source control measure
Structural BMPs	Vegetative filter strip	Transport control measure
	Terrace	Transport control measure
	Grassed swales	Transport control measure
	Detention pond	End measure
	Wet lands	End measure

**Table 2:** Implementation cost data for each BMP.

**The type of BMPs**	Cost function*	Life cycle(years)	**Maintenance cost (%)**
Wet lands	C = 30.6 V^0.71^	25	3
Detention pond	C = 24.5 V^0.69^	25	3
Grassed swales	$0.25—$0.50/ft^2^	5	10
Vegetative filter strip	$0.30—$0.70/ft^2^	2	15

*The units of V and C are ft^3^ and dollars ($), respectively.
